# *Ammopiptanthus nanus* Population Dynamics: Bridging the Gap Between Genetic Variation and Ecological Distribution Patterns

**DOI:** 10.3390/biology14020105

**Published:** 2025-01-21

**Authors:** Jingdian Liu, Mengmeng Wei, Jiayi Lu, Shiqing Liu, Xuerong Li, Xiyong Wang, Jiancheng Wang, Daoyuan Zhang, Ting Lu, Wei Shi

**Affiliations:** 1 College of Forestry and Landscape Architecture, Xinjiang Agricultural University, Urumqi 830052, China; ariiiiiink@gmail.com (J.L.); 18509976847@163.com (J.L.); li_xuerong_123@163.com (X.L.); 2 State Key Laboratory of Desert and Oasis Ecology, Key Laboratory of Ecological Safety and Sustainable, Development in Arid Lands, Xinjiang Institute of Ecology and Geography, Chinese Academy of Sciences, Urumqi 830011, China; weimengmeng21@mails.ucas.ac.cn (M.W.); lsq1998818@163.com (S.L.); wangxy@ms.xjb.ac.cn (X.W.); zhangdy@ms.xjb.ac.cn (D.Z.); 3 Turpan Eremophytes Botanic Garden, The Chinese Academy of Sciences, Urumqi 838008, China; www-1256@ms.xjb.ac.cn; 4 Xinjiang Key Lab of Conservation and Utilization of Plant Gene Resources, Xinjiang Institute of Ecology and Geography, Chinese Academy of Sciences, Urumqi 830011, China

**Keywords:** *Ammopiptanthus nanus*, endangered species, EST-SSR

## Abstract

*Ammopiptanthus nanus*, a Tertiary-era endangered plant, is of great scientific value. In this research, we focus on *A. nanus* population dynamics in an effort to bridge the divide between micro genetic variation and a macroscopic ecological pattern of distribution. The population structure of 129 wild specimens of *A. nanus* from eight populations was analyzed using EST-SSR molecular markers in this research. The Mantel test and RDA analysis have been used in this research to investigate the factors that influence the genetic diversity of *A. nanus*. Using 15 pairs of SSR primers, a total of 227 alleles were detected in 129 samples from 8 populations. The mean number of alleles was 17, and the average expected heterozygosity was 0.405. It is shown that wild *A. nanus* is divided into six individual populations. *A. nanus* are significantly affected by wind speed in terms of the variation of genetics. It is suggested that a nature conservation area for *A. nanus* be established as soon as possible, based on our results and the current natural distribution of the species. It is necessary to focus on the issue of pests and diseases while simultaneously preventing the continuation of anthropogenic woodcutting and disaster. Manual seedling collection should be employed in regions where the environment permits. Through making use of manual breeding techniques, this will contribute to the growth of the natural population of *A. nanus*.

## Simple Summary

The tertiary relict plant *Ammopiptanthus nanus* is classified as a level II endangered plant in China, whose survival is threatened by human activities and climate change. The population structure of 129 wild specimens of *A. nanus* from 8 populations was analyzed using EST-SSR molecular markers in this research. Results indicate that the genetic variation of *A. nanus* is significantly affected by wind speed, and there are 6 independent populations of *A. nanus* in the wild. This discovery underscores the importance of establishing nature reserves, developing seed banks for breeding, and monitoring pests. These measures can help prevent the decline of this species, thereby preserving our valuable biodiversity.

## 1. Introduction

Plant genetic diversity is a cornerstone of biodiversity, encompassing the spectrum of genetic variation among individuals within populations. It is not only a vital component but also the foundation of biological diversity [1]. This diversity arises from the mutations and accumulations of genetic material as species evolve, thereby shaping their capacity to adapt to environmental changes and their potential for evolutionary innovation [2]. A species with richer genetic diversity wields a stronger adaptability to its surroundings and possesses a more robust potential for evolution [3]. Consequently, this diversity plays an integral role in influencing the resilience and stability of ecosystems.

The only broad-leaved evergreen shrub in the gravelly deserts of Xinjiang, Mongolia, China, *Ammopiptanthus* is classified under the APG IV system as belonging to Fabaceae, Papilionoideae, and Tr. sophoreae. The scientific name of *Ammopiptanthus* is derived from the Ancient Greek words ἄμμoς (ámmos, meaning “sand”) and *Piptanthus*, which is the genus from which *Piptanthus* is divided. According to Mr. Zheng Sixu, *Ammopiptanthus* was distinguished from *Piptanthus* in 1959 by virtue of its alternate flowers, stipules, and petioles [4]. *Ammopiptanthus mongolicus* and *Ammopiptanthus nanus* are the sole two species of this genus in existence, and they are both ancient Tertiary relict species. *A. nanus* is even more uncommon and is primarily found in Wuqia County, Xinjiang, China, with a concentration on both sides of the Tianshan Mountains [5]. These Tertiary Fabaceae relict plants are of significant scientific value for the study of the Central Asian region’s paleogeography, paleoclimate changes, and ancient flora changes [6]. *A. nanus* originated in the ancient Mediterranean tropics, and maintains normal growth and development processes and biological and ecological characteristics of the fragile ecosystem in desert areas, and plays an important role in the maintenance of said ecosystem [7]. It is also an excellent material used as a greening ornamental and for wind and sand stabilization in arid areas. It is highly adaptable, and is able to withstand −30~50 °C temperature conditions, drought stress, salt stress, and wind and sand erosion [8]. It is also utilized as medicine by the local Kirghiz people in Wuqia County. The branches and leaves of the plant have a bitter taste and contain a variety of alkaloids that can be used for medicinal purposes. These alkaloids have the ability to dispel wind-dampness and activate blood circulation to disperse traces. Additionally, the plant can be used as an insecticide and fuel [9].

The sole broad-leaved evergreen shrub in Xinjiang is *A. nanus*. *A. nanus* is capable of thriving in the stony Gobi and extremely arid barren mountains, where it plays a critical role in improving soil, preventing winds, and fixing sands [10]. It has a large root system, good nitrogen-fixing capacity, and physiological and ecological characteristics that are well-suited to harsh habitats. It can endure stony slopes, stream scouring channels, and gravelly river rambles at an altitude of 1800–2600 m due to its well-developed root system [11]. *A. nanus* has strong economic value and use value in addition to its high ecological value and research value. It is a dominant and established species that guarantees the stability of the community structure in its arid distribution area [12]. The distribution area of *A. nanus* is located in the minority gathering area of Xinjiang, Wuqia County, where the Kyrgyz population comprises 70% of the total. The Kyrgyz are known for their traditional form of subsistence, which involves herding. In addition to serving as fuel wood for the local ethnic minorities, *A. nanus* is also highly valued as a medicinal plant. Local Uyghurs in Wuqia County, Xinjiang, also consume its flowers and seeds in addition to the stems and foliage as medicinal substances [13]. This suggests that it may exhibit low toxicity, and the medicinal value of *A. nanus* requires further rationalization and development. In the interim, the *A. nanus* community, which has a restricted global distribution, is in dire need of more rational and effective countermeasures to ensure the conservation of germplasm resources. These countermeasures should be founded on a variety of methods, including comprehensive sample collection and genetic structure analysis.

Microsatellite sequence marker is another term for simple sequence repeat (SSR) [14]. SSR molecular markers are typically comprised of tandem repeats of 1–6 bases, and the sequences are typically less than 100 bp in length. In terms of genetic marker technology, SSR molecular markers have demonstrated high polymorphism and excellent reproducibility, as they are based on PCR reactions. The primary benefits of SSR molecular markers are as follows: they exhibit simple repetition, as SSR molecular markers are composed of short sequence repeat units, which typically range from two to six base pairs; they possess high polymorphism, due to the fact that they have varying repeat numbers and combinations based on the individual; they are numerous and can encompass the entire genome, revealing a high degree of polymorphism; and they are characterized by their straightforward, rapid, and cost-effective amplification and detection processes. SSR molecular markers also show potential in the study of adaptive evolution in plants. By analyzing SSR molecular markers within a specific gene region, it is possible to investigate the level of genetic diversity and confirm the adaptation of plants to environmental stresses [15]. This information will also be beneficial and instructive for future genetic conservation efforts, given the unique ecological environment in which *A. nanus* is situated.

Our research was conducted to establish a scientific foundation for the conservation and sustainable management of *A. nanus*, a species that is naturally distributed in Xinjiang. To achieve this, we employed EST-SSR markers to assess the genetic diversity and population structure of this species, and we analyzed the results in the context of meteorology and geography.

## 2. Materials and Methods

### 2.1. Experimental Materials

**Figure 1:**
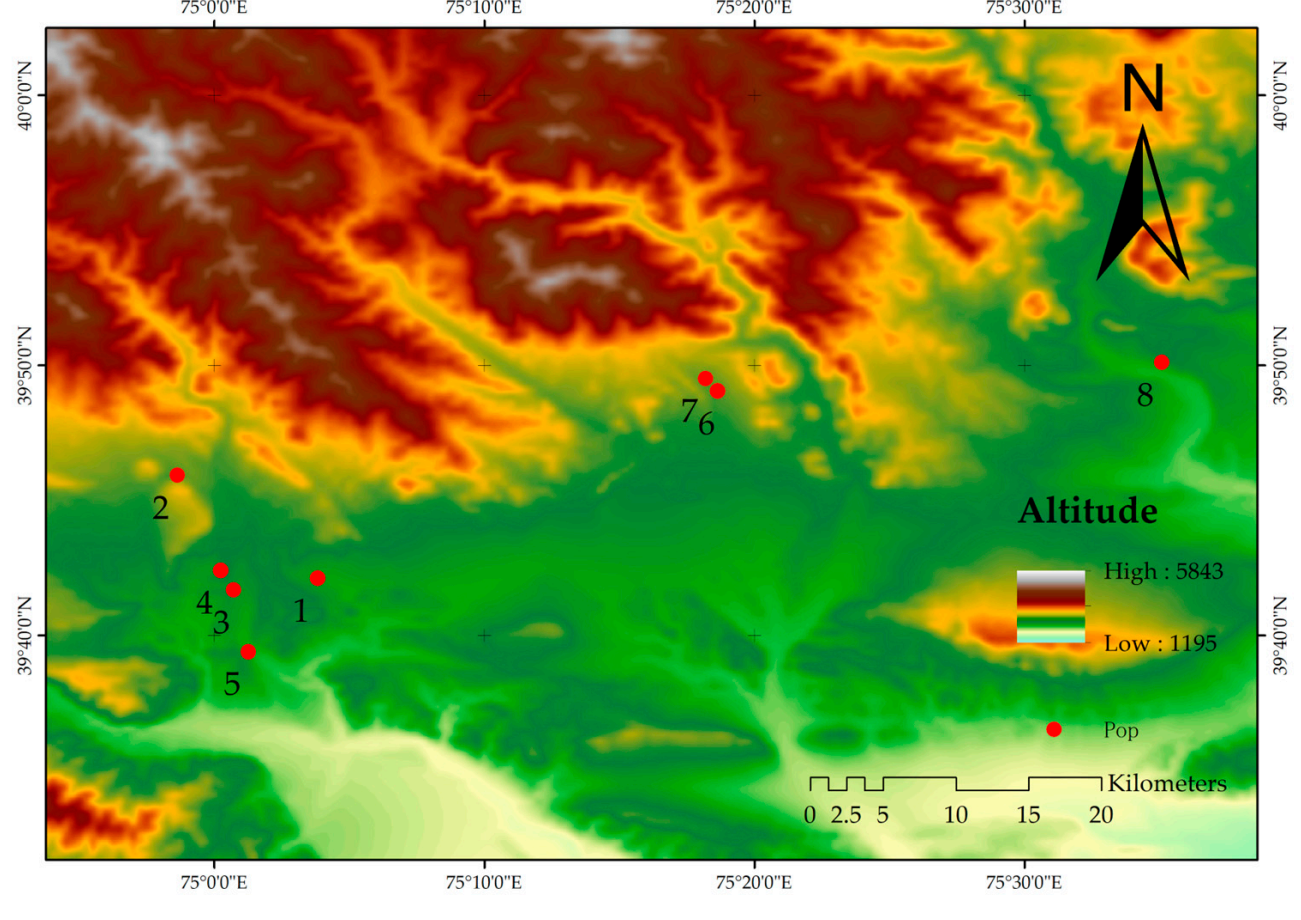
Distribution point and elevation data from 8 *A. nanus* populations.

A total of 129 samples of *A. nanus* plants were collected from eight distinct populations in this research. It is necessary to note that *A. nanus* exists in an extremely small population. The natural environment in which it is placed has emerged as harsh, and its distribution range is particularly limited. There are numerous difficulties associated with habitat surveys and sample collection. We were only able to obtain samples from eights populations because of the limited options posed by the habitat conditions. At random, healthy *A. nanus* individuals were selected from each population, and the leaves were collected and desiccated on silica gel. Subsequently, they were stored in a −20 °C refrigerator as a reserve. Environmental information was recorded for each population during the sampling procedure (Figure 1, Table 1).

### 2.2. DNA Extraction and PCR Amplification

The SSR primer sequences were derived from our previously published EST-SSR primers [16], which have been evaluated for reliability in practical applications. Utilizing the DNAsecure plant kit (Tiangen Biotech (Beijing) Co., Ltd., Beijing, China), DNA was extracted from *A. nanus* plant material in accordance with the manufacturer’s guidelines (Table 2).

The 129 *A. nanus* DNAs that were extracted were employed as templates in a 25 µL SSR-PCR reaction system. The templates employed were 1 µL of DNA template, 1 µL of upstream and downstream primers, 12.5 µL of Taq mix, and 9.5 µL of ddH_2_O. The PCR products were pre-denatured at 95 °C for 3 min, denatured at 95 °C for 15 s, annealed at 56 °C for 30 s, and extended at 72 °C for 15 s for a total of 30 cycles.

The PCR products were stored in a refrigerator that was shielded from light at a temperature of 4 °C. The PCR products were separated using agarose gel electrophoresis and stained with the nucleic acid reagent SYBR Gold Molecular Probes (Tiangen Biotech (Beijing) Co., Ltd., Beijing, China) solution. Ultimately, the Bio-rad gel imaging system was employed to capture and record the images.

Capillary electrophoresis was employed to analyze the PCR products and to conduct statistical analyses of the amplified fragment size and peak electrophoresis plots. The results were recorded in an Excel format document, and any missing values were denoted as 0. The data served as the foundation for the genetic structure analysis, genetic diversity analysis, and correlation analysis of environmental factors on genetic diversity.

### 2.3. Data Analysis

The level of gene richness and genetic diversity within the population is reflected in the average number of alleles (Na) and effective number of alleles (N) among the genetic diversity parameters. The proportion of genotypic heterozygosity in the population was evaluated using the following metrics: expected heterozygosity (He), observed heterozygosity (Ho), and unbiased heterozygosity (uHe). The Shannon Information Index (I) is a measure of the complexity of genotypes and gene frequencies within a population. Utilizing GenAlEx 6.51 software [17], we conducted an analysis of the genetic diversity of *A. nanus* populations and computed numerous population-level genetic diversity parameters in this investigation. A molecular analysis of variance (ANOVA) was conducted to disentangle the sources of genetic variance, and principal component analysis (PCoA) was employed to generate Nei genetic distances and gene flow matrices between populations. Additionally, a phylogenetic tree of *A. nanus* was generated using MEGA 11 software [18]. The following parameters were assessed: number of alleles (Na), number of effective alleles (Ne), Shannon index (I), observed heterozygosity (Ho), expected heterozygosity (He), gene flow (Nm), unbiased expected heterozygosity (uHe), fixation index (F), and index of genetic differentiation (Fst). Utilizing the allele frequency analysis function of CERVUS 3.0.7 software [19], fundamental parameters, including polymorphic information content, were assessed after the sequencing results of 15 pairs of microsatellite primers were analyzed.

Fis, Fit, and Fst are critical parameters for evaluating the extent of genetic differentiation among populations. The Fst index, which is the most frequently employed index, is capable of accurately representing the heterozygosity of alleles in a population. The genetic structure of the populations is entirely consistent and there is no genetic differentiation when the value of Fst is 0, whereas when the value of Fst is 1, it indicates that there is complete differentiation among the populations. In general, the genetic differentiation among populations is not obvious and can be disregarded when the value of Fst is between 0 and 0.05. The genetic differentiation among the populations is moderate when the value of Fst is between 0.05 and 0.15. The genetic differentiation among the populations is large when the value of Fst is between 0.15 and 0.25. The genetic differentiation among the populations is very large when the value of Fst is greater than 0.25.

The software Bottleneek v 1.2.02 [20] was employed to determine whether *A. nanus* populations had recently encountered bottleneck effects. The two-phased model of mutation (TPM) was selected as the computational model. In order to identify *A. nanus*, the Wilcoxon marking test and the sign test were implemented. The assay’s accuracy was guaranteed by setting the number of calculations to 10,000 [21]. This was further enhanced by examining whether the heterozygosity excess was substantial within each population and whether it experienced a bottleneck effect using a graphical representation of pattern migration.

A total of 11 topographic variables (Table 3) and 25 climatic variables (Table 4) were chosen as alternative variables for the model, with the climatic variables being sourced from the WorldClim database [22], on the basis of the field survey and historical literature references [23]. The elevation, slope, and slope direction variables were determined from the 30 m resolution DEM data that were obtained from the Geospatial Data Cloud Platform of the Chinese Academy of Sciences Computer Network Information Center (CNIC) [24]. The distance to rivers was calculated using Euclidean distance by downloading 1:1 million national river data, and the elevation, slope, and slope direction variables were calculated using DEM data with a 30 m resolution downloaded from the Geospatial Data Cloud Platform of the Computer Network Information Center of the Chinese Academy of Sciences (CNIC). The Normalized Difference Vegetation Index (NDVI) was adopted from the 30 m yearly maximum NDVI data in China from 2000 to 2020, produced by the remote sensing team of land use and global change at the Institute of Geographic Sciences and Resources of the Chinese Academy of Sciences (IGRS) [25].

The 36 environmental variable layers’ raster size, unified coordinate system, and layer boundaries were unified and cropped in ArcGIS V 10.8 software in accordance with the administrative boundaries of the Xinjiang Region. The autocorrelation analysis of the 36 variables was conducted using the R package “vif” to eliminate the 16 variables with high correlation and retain the other 20 variables for the subsequent analysis [26].

Mantel tests permit assessment of the correlation between genetic differentiation and geographic distance and the analysis of multivariate data [27]. Redundancy analysis is primarily pertinent to the analysis of linear relationships and can effectively manage and analyze complex datasets that contain a large number of genetic markers and environmental predictors. Two methods of analysis were employed to examine the correlation between environmental factors and genetic diversity.

The genetic diversity of Rough Stemmed Bully in the four main deserts was correlated with each environmental factor using the R package “linkET” in the Mantel test [28]. The R package “ggplot2” was employed to generate the graphs [29]. The statistical significance of the edges was indicated by the hue, while the width of the edges corresponded to the Mantel’s r statistic for the corresponding distance correlation. The genetic diversity index was selected as the genetic factor for the redundancy analysis, alongside climatic data and geographic data (latitude and longitude of the sampling site). The R package “vegan” was employed to conduct a redundancy analysis (Table 5) [30].

## 3. Results

### 3.1. Genetic Diversity Analysis of A. nanus

Using 15 pairs of SSR primers, an analysis of 129 samples collected from eight populations was conducted. The results indicated that the 15 primer pairs contained a total of 227 alleles, and the number of alleles varied substantially across the loci, ranging from a minimum of 3 (F14 and F15) to a maximum of 45 (F4). These loci were highly polymorphic in the population, as evidenced by the mean number of alleles, which was 17. The majority of the loci exhibited a high level of total genetic diversity, with a mean value of 0.503 and a range of total genetic diversity (Ht) from 0.007 (F15) to 0.957 (F4). This indicates that the population had a significant level of genetic diversity. The expected heterozygosity (He) is indicative of the anticipated frequency of heterozygotes in the population, and a high level of genetic diversity was exhibited, with a mean value of 0.405. The observed heterozygosity (Ho), which is the actual observed frequency of heterozygotes, had a mean value of 0.232. This value is typically lower than the expected heterozygosity, which may be attributed to heterozygous deletions caused by inbreeding or genetic drift.

The value of Fis is indicative of the population’s degree of heterozygote deletion within the population. The average Fis value was 0.586, which suggested that heterozygous deletions were present in the majority of the loci. Positive values are indicative of heterozygous deletions. The average fit value was 0.658, which suggested that there was a degree of genetic differentiation among populations. The average Fst value was 0.199, which indicated that the differentiation among populations accounted for a proportion of the total genetic variation. This value is used to characterize the proportion of genetic variation among populations. The Nm value is a metric that is employed to estimate gene flow and represents the number of individuals that migrate between populations. The average effective number of migrants was 2.196, which may suggest that there exists a certain amount of genetic exchange between populations. The PIC value is employed to quantify the polymorphism of genetic markers, with higher values suggesting that the genetic markers are more beneficial. The average PIC value was 0.509, suggesting that these markers have a higher level of information content for genetic analysis (Table 6).

The extent of genetic variation was demonstrated by the fact that the number of effective alleles (Na) varied from 1.667 (Pop 2) to 9.467 (Pop 6) in the eight populations that were collected. In the field of population genetics, the effective population size (Ne) is a critical parameter that reflects the genetic diversity of the population and the impact of genetic drift. The Ne values varied from 0.347 (Pop 2) to 1.468 (Pop 6). The Shannon Information Index (I) is a metric that reflects the diversity of allelic variants within a population, with values ranging from 0.543 (Pop 4) to 1.203 (Pop 7). The proportion of heterozygotes that are genuinely observed in a sample is known as observed heterozygosity (Ho). The values of Ho for different populations ranged from 0.134 (Pop 8) to 0.389 (Pop 6). The expected proportion of heterozygotes (He) is determined by the allele frequencies. It ranged from 0.292 (Pop 4) to 0.578 (Pop 6). Another measure of the expected proportion of heterozygotes is unweighted expected heterozygosity (uHe), which ranged from 0.333 (Pop 4) to 0.595 (Pop 6). The informational value of genetic markers in population genetics investigations is quantified by polymorphic information content (F), which ranged from 0.355 (Pop 4) to 0.754 (Pop 7). The percentage of private alleles (PPB) is the proportion of alleles that are exclusive to a specific population. The PPB values for the studied populations ranged from 33.33% (Pop 2 and Pop 3) to 100.00% (Pop 7). Particularly, the mean value of 74.17% suggests that the diverse natural populations of *A. nanus* possess more exclusive alleles. The genetic adaptability and uniqueness of the populations may be suggested by the presence of private alleles and genetic differentiation among populations. Special conservation measures may be necessary to preserve the genetic diversity of populations with high PPB values. Furthermore, the presence of higher He and uHe values suggests that these populations possess a greater degree of genetic variation, which is crucial for the long-term survival of the population and the facilitation of adaptive evolution (Table 7).

### 3.2. Genetic Relationship and Population Structure Analysis

**Figure 2:**
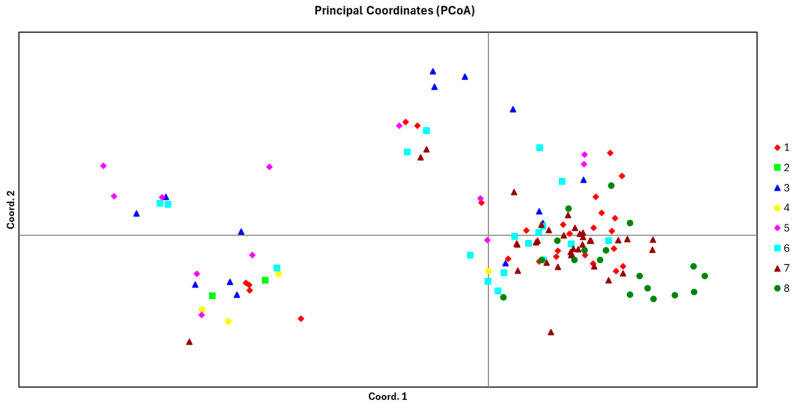
Principal coordinate analysis of 129 *A. nanus* base on genetic distance.

Principal Coordinate Analysis (PCoA) is used to analyze and illustrate the similarities or distances between individuals or populations. Figure 2 displays the PCoA results for the 129 studied *A. nanus* samples. The genetic distances between populations can be estimated by examining the differences in distributions on the axes. The PCoA can be used to observe the genetic clustering of various populations. The eight populations of *A. nanus* were not clearly classified, but they exhibited a certain degree of regularity, which is representative of a local clustering trend. Populations 1, 6, 7, and 8 exhibited clustering.

**Figure 3:**
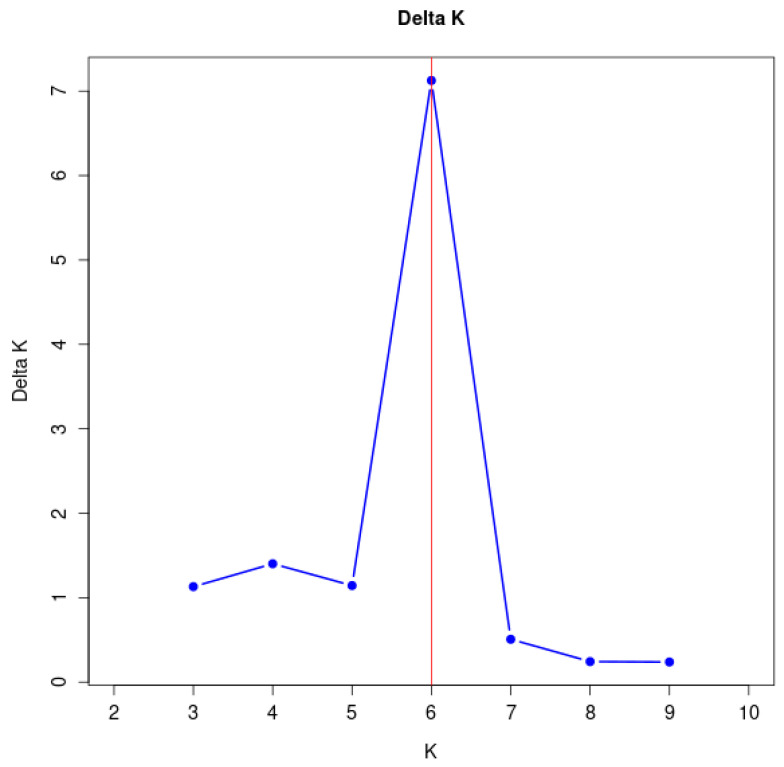
Bayesian model-based genetic structure analysis of *A. nanus*. The blue dots show the Delta K values corresponding to different K values. The red vertical line marks the peak of Delta K at K = 6.

**Figure 4:**
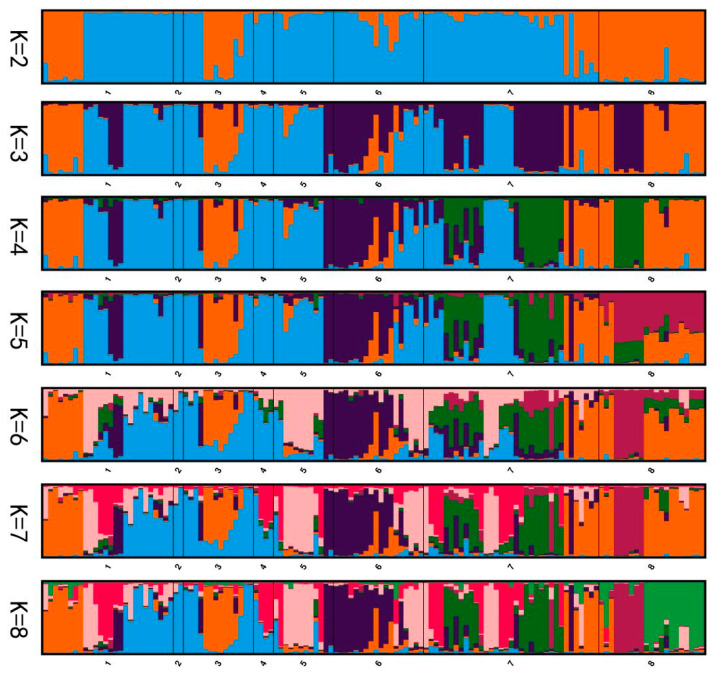
Ancestral clustering for K = 2 to K = 8. Each *A. nanus* individual is expressed as a line segment, where black lines are used to distinguish from 8 different populations. Different colors represent different clustering groups. For example, at K = 2, there are only two colors (e.g., blue and orange), indicating that all individuals are divided roughly into two clustering groups. When K = 3, a third color (e.g., purple) is present, which indicates that individuals are divided into three clustering groups, and so on.

On the other hand, Structure Harvester was used to evaluate the population’s genetic composition. The number of groupings is denoted by K in Bayesian cluster analysis. The population is most likely to be divided into six genetically relatively independent groups, as our calculation yielded K = 6 (Figure 3). The structure clustering plot from K = 2 to K = 8 indicated that the populations of *A. nanus* exhibited various levels of genetic exchange (Figure 4).

**Figure 5:**
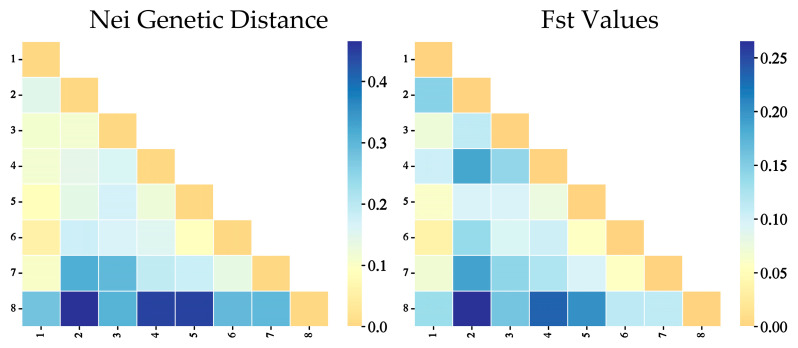
Pairwise population matrix of Nei genetic distance and Fst values of *A. nanus* populations.

Nei genetic distance (Table 8) and Fst values (Table 9) are measures of genetic differences between populations. Differences in allele frequencies are the basis for their computation. It is feasible to assess the critical parameters of genetic disparities and population similarity through their utilization. Based on these two matrices, we discovered that the genetic distance between Pop 6 and Pop 1 was the smallest, suggesting that they are genetically the least distinct. The genetic distance between Pop 2 and Pop 7 was the greatest, suggesting that they are the most genetically distinct. The relatively large genetic distance between Pop 8 and all other populations suggested that it is genetically distinct from the other populations in a substantial manner (Figure 5).

**Figure 6:**
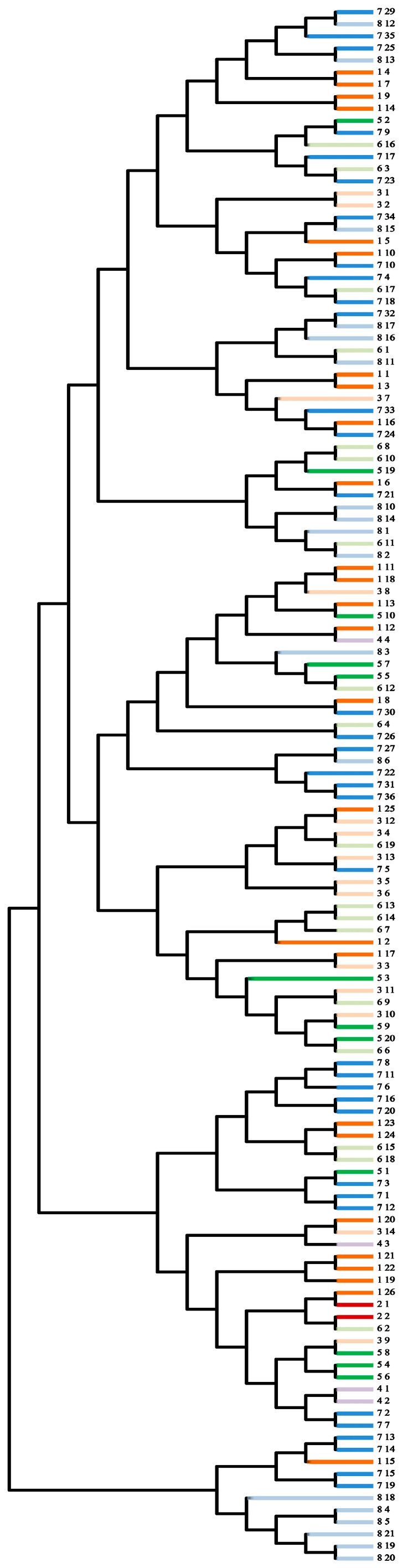
Phylogenetic tree of 129 *A. nanus* samples. Different colors represent different populations.

The Neighbor-Joining (NJ) method was employed to construct phylogenetic trees between 129 *A. nanus* samples using MEGA11 v11.0.13 software. The evolutionary relationships between individuals were investigated by minimizing the sum of the distances between all nearest neighbors in the tree. The results were also consistent with those of previous PCoA and structure analyses. Each population contained a certain degree of intermixing (Figure 6).

AMOVA analyses enabled a more precise evaluation of the extent of genetic differentiation among various populations of *A. nanus* and the determination of its percentage of the total genetic variance (Table 10). Only 1% of the variation in the 129 samples collected from the eight populations was attributed to population differences. Within-population variation accounted for the overwhelming majority (99%) of the variation. Between-individual variation accounted for up to 77% of the variation, while intra-individual variation accounted for an additional 22%.

The Wilcoxon’s signed-rank *p*-value test demonstrated that the TPM model maintained a “L” distribution pattern for all eight populations of *A. nanus*. This suggests that the genetic structure of *A. nanus* is comparatively stable and has not been subjected to any bottleneck effects in the recent past (Table 11).

### 3.3. A. nanus Relationship Between Genetic Diversity and Environmental Factors

**Figure 7:**
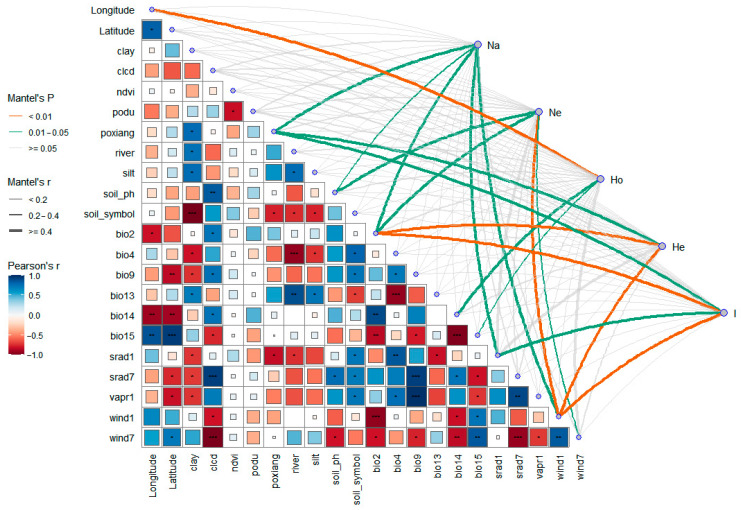
Mantel test of *A. nanus* based on SSR molecular markers. An asterisk (*) indicates statistical significance: * represents statistical significance at the 95% confidence level (*p*-value < 0.05), ** at the 99% confidence level (*p*-value < 0.01), and *** at the 99.9% confidence level (*p*-value < 0.001).

To explore the associations between environmental factors and genetic diversity in *A. nanus*, we employed the Mantel test to evaluate the correlation between genetic differentiation and geographic distance, and explore evolutionary hypotheses and analyze multivariate data. The genetic diversity index of *A. nanus* was correlated with SSR molecular markers and environmental factors, as evidenced by the Mantel test results. A significant correlation was observed between observed heterozygosity (Ho) and longitude, suggesting that geographic location may be a significant factor in the regulation of Shannon Information Index in *A. nanus* populations. It was also found that anticipated heterozygosity (He) and Shannon Information Index (I) would exhibit a significant correlation with the daily mean temperature range. In January, wind speed exhibited significant correlations (*p* < 0.01) with effective population size (Ne), expected heterozygosity (He), and Shannon Information Index (I) (Figure 7).

**Figure 8:**
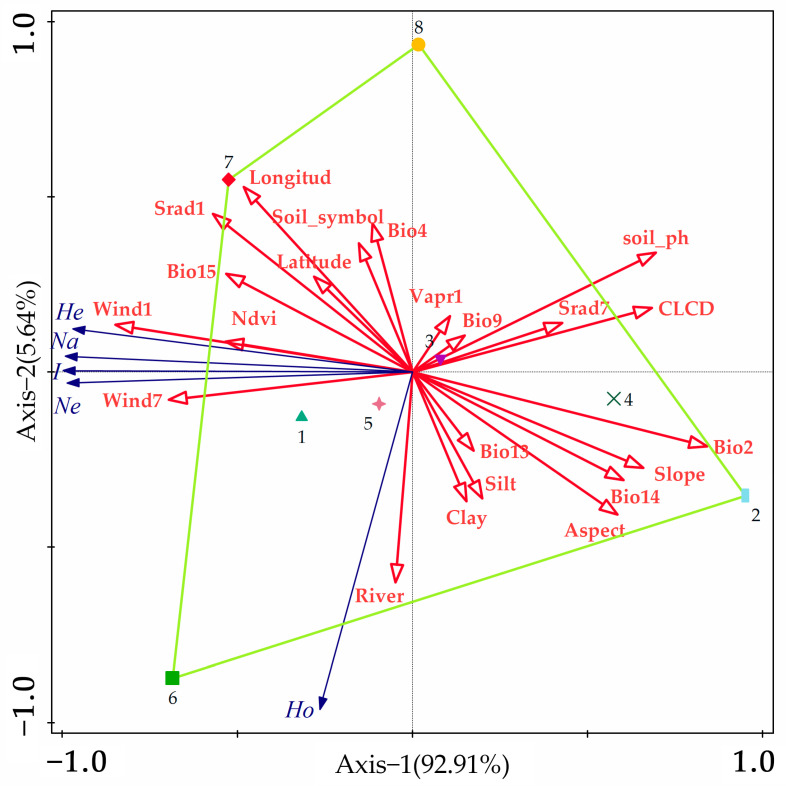
Redundancy analysis of *A. nanus* based on SSR molecular markers. On the horizontal axis, 92.91% of the data variance is explained, while on the vertical axis, 5.64% of the variance is revealed.

In order to investigate the direct and indirect relationships between variables, we also used redundancy analysis to explore the association between genetic variation and specific environmental factors. The results of RDA analysis showed that wind speed had a very significant effect on genetic variation in *A. nanus*, which was similar to the results of the Mantel test. The other sections also showed that longitude, solar radiation, precipitation, and vegetation cover all had an effect on genetic variation (Figure 8).

**Figure 9:**
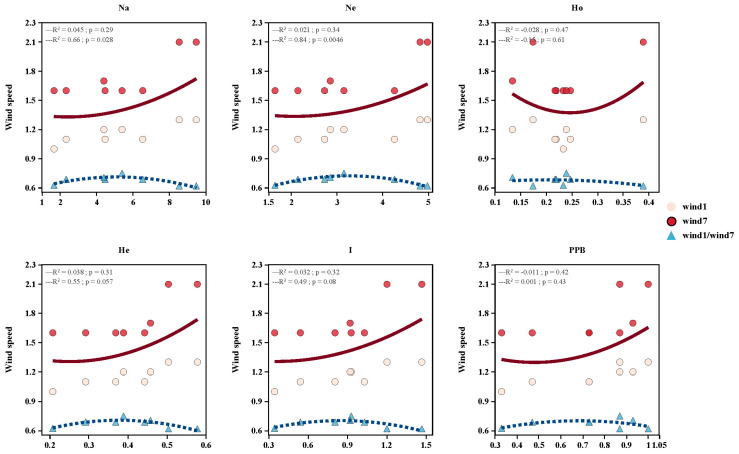
Bilinear analysis of the effect of wind speed on genetic diversity in *A. nanus*.

In order to find out more about the effect of wind speed on the genetic diversity of *A. nanus*, we used bilinear analysis to create Figure 9. The figure includes six sub-figures that show the association between wind speed and different genetic diversity indicators.

The figure shows that *Na* revealed a certain upward trend as wind speed increased. The fitted curve of wind speed (red points) in July shows a more significant positive correlation (*p* = 0.028). Ne showed a similar signal; however, the correlation was relatively weak (*p* = 0.046). The relationship between Ho and wind speed was more complicated. The fitted curve of wind speed in July had a trend of increasing and then decreasing, as well as a trend of increasing and then decreasing. The correlation between wind speed and Ho in January was not significant (*p* = 0.47), but the fitted curve for wind speed in July exhibited an increasing and then decreasing trend. On the one hand, the relationship between Ho and wind speed was more complicated. On the other hand, He and I showed a trend toward higher wind speed, though the correlation was not particularly strong.

These findings give more proof supporting the results we reached based on the Mantel test and RDA analysis. The genetic diversity of *A. nanus* was significantly influenced by wind speed, and the response of various genetic diversity indicators to wind speed varied accordingly. This supplies more intuitive evidence to help with an expanded understanding of the process in which environmental factors affect the genetic structure and diversity of *A. nanus*.

## 4. Discussion

The significance of genetic diversity in the preservation of habitat diversity and species diversity is now widely acknowledged [31]. Additionally, genetic diversity is acknowledged as a critical element of species resistance and ecosystem resilience. Modern molecular biology has provided us with a greater number of methods to acquire information [32]. Molecular marker technology is considered one of the most effective bases for determination in the conservation and identification of rare and endangered plants [33]. The utilization of EST-SSR molecular markers to examine the genetic diversity and structure of *A. nanus* will not only facilitate our comprehension of the mechanism of endangerment of *A. nanus*, but also serve as a valuable genetic foundation for the development of conservation strategies and management units.

It is obvious that the genetic diversity of *A. nanus* is closely linked with its endangerment process when the historical research is compared to the genetic diversity indicators in the present study [34]. Genetic differentiation and diversity loss have been severely exacerbated by habitat fragmentation, which has impeded gene exchange between populations. This not only restricts the adaptive evolution of populations but also elevates the likelihood of harmful recessive gene purity [35]. Conducting a comprehensive examination of this association will enable us to identify the primary factors that are responsible for the decrease in their genetic diversity [36]. The scientific foundation for the development of effective conservation measures, such as the establishment of ecological corridors to facilitate gene flow and the protection of critical habitats to maintain population size, will be established by identifying the specific gene loci or genetic processes in *A. nanus* that have been negatively impacted by human activities or environmental changes in order to ensure the continued existence of *A. nanus* and to reverse the decline in its genetic diversity.

Unfortunately, *A. nanus* is endangered as a consequence of the disaster of its natural habitat, human disturbance, and the limited extent of its range [37]. This has led to a decrease in populations and an elevated risk of genetic drift and inbreeding [38]. Research shows that endangered species are frequently associated with diminished genetic diversity, which renders them more susceptible to environmental changes and increases the susceptibility of populations to threats such as disease and climate change [39]. In the instance of *A. nanus*, the absence of genetic diversity may result in small populations struggling to produce sufficient genetic variation to deal with new environmental challenges, including altering precipitation patterns and increasing temperatures. Therefore, genetic diversity research is essential for the evaluation of the extent of endangerment and the development of scientific conservation strategies. Comprehending the current level of genetic diversity will enable potential genetic risks to be identified in a timely manner, and targeted measures can thereby be implemented to prevent further loss of genetic diversity [40].

*A. nanus*, an ancient relic species from the Tertiary period, has undergone a protracted period of geological and climatic changes. This antiquity has allowed it to accumulate distinctive genetic information [41]. It has adapted to the complex and variable environment of Central Asia throughout its evolutionary voyage. The genetic material of *A. nanus* frequently exhibits a protracted process of adaptation, which leads to specific gene combinations and allele distribution patterns [42]. Investigation of the genetic diversity of *A. nanus* will contribute to the understanding of its mechanisms of conservation and the evolution of its archaic genetic information. Additionally, it will facilitate comprehension of which distinctive genetic characteristics have been preserved and enhanced through extended periods of natural selection. The findings of this significant investigation may also offer valuable insights into the evolutionary trajectory of relict plants in the ancient Mediterranean. The genetic underpinnings of *A. nanus*’s adaptations to its primordial environment can be elucidated through the examination of the diversity of wild populations. This analysis will also establish a foundation for predicting the species’ capacity to adapt to future environmental changes.

In our previous research on *A. nanus*, we analyzed the genetic diversity of *A. nanus* under the condition of translocation conservation using transcriptome sequencing, established EST-SSR markers, and analyzed the efficacy of ex situ conservation in *A. nanus*. Our findings indicate that the current ex situ conservation of *A. nanus* requires reinforcement, and the majority of the germplasm resources are not included in the translocation conservation [22]. However, the genetic structure and diversity of *A. nanus*’s wild populations have not been thoroughly investigated. In order to ensure the preservation of endangered species, it is imperative that we assess the prospective genetic diversity of populations [43]. Genetic diversity is frequently weakened in threatened or range-restricted species, which requires analysis of the genetic structure and diversity of their natural populations.

ISSR molecular markers have been used to measure the genetic diversity and differentiation levels of 120 materials from six populations of *A. nanus* in previous research [44]. The results showed that there were significant barriers to gene flow among populations, high genetic divergence among populations, and high genetic similarity among individuals within *A. nanus* populations. We discovered that the genetic diversity of *A. nanus* have decreased gradually over time, which suggests that the unique genetic resources of *A. nanus* are decreasing. This exhibits a significant challenge for endangered and protected plants with limited distribution ranges. The present research is more representative of the overall genetic status of *A. nanus* and has a wider coverage than previous studies, which have had limited sample collections. EST-SSR markers are highly variable and highly accurate, which can more accurately supply genetic differences within and among populations in the context of research methodology. The polymorphism of various loci was elucidated, and 227 alleles were identified by analyzing 15 pairs of EST-SSR primers in this research.

It is interesting to note that when all existing *A. nanus* populations were evaluated for genetic diversity in this study using EST-SSR, genetic differentiation between populations was not significant, and the main genetic differences existed between individuals. This may stem from the conserved nature of expressed sequence tags, and the genetic similarity shown in *A. nanus* for adaptation to microclimates. Bottleneck analyses also showed this, with *A. nanus* having a relatively stable genetic structure.

Morphology and essential ecological surveys were the constraints that have limited previous investigations, making them incapable of supplying information about the potential genetic diversity and population structure of the species [45]. Our research was able to capture more subtle genetic differences among populations as a result of the expansion of the sample range, which is necessary for the development of precise conservation strategies. The conclusions of genetic analyses indicate that our survey identified only six populations of *A. nanus* in the wild, and the natural population differentiation of *A. nanus* appears to be limited. This may be due to the longer lifespan of individuals and a lower turnover of offspring. The results of this study suggest that special conservation measures, such as increased captive breeding and gene introgression, can be applied for enhancing genetic diversity in populations with low genetic diversity.

The establishment of nature reserves is of great significance as it can provide these populations with a relatively stable living environment, effectively reducing external disturbances and preventing further isolation and loss of genetic diversity that may result from habitat destruction and human activities [46]. For Population 2, which exhibited low genetic diversity, the establishment of a protected area is crucial for safeguarding its existing gene pool. This could prevent the loss of its unique alleles due to habitat fragmentation or excessive human exploitation, thereby maintaining its survival and reproduction capabilities in the natural environment and facilitating the natural recovery of the population and gene exchange.

From the angle of population propagation, *A. nanus* is confronted with difficulties in the form of a lack of natural regeneration and a declining population size in the wild [47]. Notably, the effective population size of certain populations is rather small. For instance, the effective population size of Population 2 was found to be only 0.347. Manual breeding collection strategies can play a vital role in increasing the population size and expanding the gene pool. During the seed collection process, particular attention should be paid to collecting seeds from diverse populations to fully capitalize on the genetic differences among them and enhance the genetic diversity of the offspring. For example, seeds from populations with high genetic diversity, such as Population 6, can be rationally mixed with those from other populations for breeding purposes. This approach can introduce novel gene combinations, improve the adaptability of the entire species to environmental changes, mitigate the genetic risks associated with inbreeding, and enhance the survival and vitality of the populations [48].

Multiple points of observation can be established between different populations to improve gene flow. When samples are collected on a regular basis for genetic analysis, the genetic data from different periods can be compared to evaluate the direction and intensity of gene flow. At the same time, ecological corridors are established between populations, and the corridors are planted with companion plants that *A. nanus* prefers in order to establish favorable conditions for gene exchange. Significant emphasis should be placed on the choosing of individuals with representative genotypes, excellent health, and a diverse genetic history for artificial breeding. In order to avoid inbreeding, the breeding group’s size should be limited during the reproductive process. Breeding regions should be chosen in locations with climatic conditions that are equivalent to *A. nanus*’s native habitat. Regular health exams and growth records of breeding individuals should be conducted by professional conservationists, and the systematic development of captive growth should be ensured. The overall feasibility and efficacy of conservation measures should be improved.

Resequencing technology will be employed to screen the primary germplasm resources of *A. nanus* in the following stage of our research. Through the resequencing analysis of *A. nanus* in a number of ecological environments and geographic regions, we will establish the ability to perform an extensive review of the genetic differentiation mechanisms and the evolutionary history of its populations. A detailed genetic map of *A. nanus* can be created based on the significant genetic data obtained from resequencing. This offers empirical evidence to support the scientific development of the biological evolution theory. These main germplasm resources will serve as valuable resources for the subsequent conservation and utilization of *A. nanus*, which guarantees the species’ future viability and evolutionary potential.

Regarding natural factors, it has been determined that wind speed exerts an extremely significant influence on the genetic variation of *A. nanus*. Additionally, longitude, solar radiation, precipitation, and vegetation coverage also have notable effects. In the process of conservation, areas characterized by relatively suitable wind speed, favorable climatic conditions, and good vegetation coverage should be selected as key conservation and restoration zones. During vegetation restoration efforts, it is essential to create a microenvironment conducive to the growth and reproduction of *A. nanus*. This can help to reduce the continuous impact of adverse environmental factors on its genetic diversity and assist in maintaining a stable genetic structure and high adaptability [49].

It is important to note that although our study did not directly investigate the impacts of pests and diseases on the genetic diversity of *A. nanus*, considering its small population size and the endangered status of some populations, any infestation by pests and diseases could potentially cause severe damage to its genetic diversity [50]. Therefore, the establishment of a pest and disease monitoring system is of utmost importance. This system should be capable of promptly detecting and effectively dealing with such problems to protect the integrity and genetic stability of its populations. Regular inspections of the health status of *A. nanus* plants and the utilization of environmentally friendly pest control methods, such as biological control or low-toxicity pesticides, are necessary to ensure the healthy growth of populations and avoid sudden population declines and genetic information loss due to pests and diseases [51].

International cooperation holds great promise for expanding genetic resources through the introduction of seed resources from different populations in other countries [52]. Given the endangered status of *A. nanus* and its limited genetic resources, international cooperation can introduce the genetic resources of *A. nanus* from regions like Kyrgyzstan to enrich its gene pool [53]. By collaborating with international research institutions, sharing research findings and genetic materials, and learning advanced conservation techniques and experiences, novel ideas and methods for the conservation of *A. nanus* can be developed. This could enhance its ability to cope with environmental changes and genetic risks and promote its status and role in global biodiversity conservation [54].

## 5. Conclusions

The genetic diversity and population structure of *A. nanus* were accurately analyzed using EST-SSR markers in this research, and the effects of environmental factors were investigated. Results showed that *A. nanus* populations exhibited a significant degree of genetic diversity, as evidenced by the identification of 227 alleles by 15 pairs of SSR primers and the high diversity at each locus. However, the observed heterozygosity was lower than the projected heterozygosity. Eight populations could be classified into six relatively independent groups, with a certain proportion of inter-population differentiation. The intra-population variation accounted for the majority of the total variance, and the genetic structure was found to be relatively stable in the recent past. Also, longitude, solar radiation, precipitation, and vegetation cover, in addition to wind speed, were found to significantly affect genetic variation in *A. nanus*.

These research findings lay a solid foundation for subsequent conservation work, enabling it to be carried out precisely. By integrating multiple strategies, the genetic resources and ecological niche of *A. nanus* can be effectively protected, contributing to the stable continuation of the *A. nanus* population and the maintenance of ecological balance. The protection of genetic diversity and the reduction of external disturbances can be accomplished through the establishment of nature reserves. The genetic diversity of the population can be expanded through the setting up of an artificial reproduction collection strategy. The impact of unfavorable environmental factors can be lessened by selecting suitable places for focused conservation and restoration. Population health and genetic stability can be conserved through the use of pest and disease monitoring systems. In the meantime, the collection of genes can be expanded through international cooperation that includes the sharing of genetic resources and other methods. This will enhance the status of *A. nanus* in the conservation of global biodiversity. Future research may expand the sample range and carry out a deeper investigation of the specific mechanisms by which environmental factors effect genetic diversity. This will offer a more comprehensive scientific basis for the sustainable government and conservation of *A. nanus*.

## Figures and Tables

**Table 1:** A detailed distribution point and elevation data from different *A. nanus* populations.

Pop	Latitude	Longitude	Elevation
1	75.063755	39.701913	2157
2	74.977305	39.765373	2464
3	75.011993	39.694605	2200
4	75.004093	39.706762	2225
5	75.021193	39.656512	2093
6	75.310487	39.817338	2453
7	75.303393	39.825023	2485
8	75.584727	39.835007	2183

**Table 2:** EST-SSR primers sequence information for *A. nanus*.

SSR Primer Pair	SSR Primer Sequence (5′-3′)	Fluorescence Tag
c11315	F:ACTGCACCTGTCCAGATTCC	5′HEX
	R:TTGATTCGCTGTCTCCCTCT	
c23139	F:AGCATGCATGTGCCTTTTTA	5′HEX
	R:ATCGGGAGAGCAACAGTACG	
c23483	F:AACGCGTCCCATTCTCTCT	5′HEX
	R:TCATCTTTCAAAAGGGGCAC	
c23540	F:GATCAGCTTCTCACCCGAAG	5′HEX
	R:TCTGGGTCTCTTGGCCTCTA	
c24361	F:AATGCATGATTGATTGTCACTG	5′HEX
	R:TTGCGGACTGCAACAATTAG	
c24982	F:CCGTCTTGCAGGTTTCAAGT	5′HEX
	R:TTACCGGCCATAGATTGAGG	
c32449	F:TCAATCTTCGTAGCTTTCCCA	5′HEX
	R:GCTCCAAAGTCCACCACATT	
c34776	F:CCACTTTGGCTCATTGCTTT	5′HEX
	R:CTGAAGGGAGTGTGGCATTT	
c34960	F:GACCCATCAAAACATGACCC	5′HEX
	R:GGACCTCAGGATGGTCAAGA	
c35322	F:TTGTTACAAAAGCCGATCCC	5′HEX
	R:TAGGAATGACTCCCACTGCC	
c44083	F:TGAAGAGACGCAATGGTGAG	5′HEX
	R:ATGCCCAACTTGGATAGTGC	
c44298	F:AGCTGTCTTCCCAGTACCGA	5′HEX
	R:ATGGTGAGCACAACCATGAA	
c44674	F:TAATTGACCTCGAACCTCCG	5′HEX
	R:TGGAGGAGAAGGGGGTTACT	
c52855	F:GAAAGGTTGGGGATTTTGGT	5′HEX
	R:AGTGGGTGAGGGTGAAACTG	
c52997	F:CAATATCCAAAGGTGGGGTG	5′HEX
	R:GCTACGTCTCGTGCTAGCCT	

**Table 3:** Topographical factor information.

Factor Code	Unit	Describe
Altitude	m	Altitude (above sea level)
Slope	°	Slope of the earth’s surface
Aspect	-	Aspect of the earth’s surface
Soil_ph	-	PH value of soil (×10)
Soil_symbol	-	Type of soil
Sand	%	Percentage of sand soils in the soil
Silt	%	Percentage of silt soils in the soil
Clay	%	Percentage of clay soils in the soil
CLCD	-	China Land Cover Dataset
NDVI	-	Normalized Difference Vegetation Index
River	-	Euclidean distance from the river

**Table 4:** Bioclimatic factor information.

Factor Code	Unit	Describe
Bio_1	°C	Annual Mean Temperature
Bio_2	°C	Mean Diurnal Range (Mean of monthly (max temp–min temp))
Bio_3	ratio	Isothermality (BIO2/BIO7) (×100)
Bio_4	standard deviation	Temperature Seasonality (standard deviation ×100)
Bio_5	°C	Max Temperature of Warmest Month
Bio_6	°C	Min Temperature of Coldest Month
Bio_7	°C	Temperature Annual Range (BIO5-BIO6)
Bio_8	°C	Mean Temperature of Wettest Quarter
Bio_9	°C	Mean Temperature of Driest Quarter
Bio_10	°C	Mean Temperature of Warmest Quarter
Bio_11	°C	Mean Temperature of Coldest Quarter
Bio_12	mm	BIO12 = Annual Precipitation
Bio_13	mm	Precipitation of Wettest Month
Bio_14	mm	Precipitation of Driest Month
Bio_15	coefficient of variation	Precipitation Seasonality (Coefficient of Variation)
Bio_16	mm	Precipitation of Wettest Quarter
Bio_17	mm	Precipitation of Driest Quarter
Bio_18	mm	Precipitation of Warmest Quarter
Bio_19	mm	Precipitation of Coldest Quarter
Srad_01	kJ m^−2^ d^−1^	Solar radiation in January
Srad_07	kJ m^−2^ d^−1^	Solar radiation in July
Wind_01	M·s^−1^	Wind speed in January
Wind_07	M·s^−1^	Wind speed in July
Vapr_01	kPa	Water vapor pressure in January
Vapr_07	kPa	Water vapor pressure in July

**Table 5:** VIFs of the remained variables.

Serial Number	Variables	VIF
1	Clay	2.044992
2	CLCD	2.32225
3	NDVI	2.553379
4	Slope	2.337304
5	Aspect	1.008888
6	River	1.77
7	Silt	2.329704
8	Soil_ph	1.288701
9	Soil_symbol	2.343339
10	Bio_13	6.116687
11	Bio_14	5.201887
12	Bio_15	9.157776
13	Bio_16	5.16566
14	Bio_17	4.412978
15	Bio_18	7.540101
16	Bio_19	7.859912
17	Bio_20	7.236885
18	Bio_21	8.331089
19	Bio_22	5.92738
20	Bio_23	5.764699

**Table 6:** Statistical values of microsatellite markers in 129 samples of *A. nanus*.

Locus	Na	Ht	Mean He	Mean Ho	Fis	Fit	Fst	Nm	PIC
F1	28	0.914	0.857	0.669	0.220	0.268	0.062	3.758	0.914
F2	16	0.319	0.279	0.067	0.758	0.789	0.128	1.707	0.39
F3	32	0.853	0.563	0.670	−0.190	0.215	0.340	0.486	0.824
F4	45	0.957	0.865	0.576	0.335	0.398	0.096	2.358	0.964
F5	36	0.920	0.829	0.644	0.223	0.299	0.099	2.287	0.918
F6	6	0.148	0.133	0.017	0.870	0.883	0.100	2.257	0.166
F7	10	0.466	0.306	0.051	0.835	0.891	0.342	0.480	0.444
F8	18	0.561	0.411	0.099	0.759	0.823	0.267	0.685	0.527
F9	10	0.315	0.239	0.076	0.680	0.757	0.242	0.782	0.351
F10	31	0.847	0.743	0.452	0.391	0.466	0.123	1.786	0.859
F11	7	0.161	0.142	0.028	0.803	0.827	0.118	1.862	0.203
F12	6	0.377	0.272	0.014	0.947	0.962	0.278	0.649	0.365
F13	4	0.505	0.315	0.105	0.666	0.792	0.376	0.414	0.44
F14	3	0.189	0.115	0.000	1.000	1.000	0.393	0.385	0.252
F15	3	0.007	0.007	0.004	0.489	0.499	0.019	13.048	0.015
Mean	17	0.503	0.405	0.232	0.586	0.658	0.199	2.196	0.509
SE	3.579	0.084	0.076	0.072	0.086	0.070	0.032	0.816	0.080

**Table 7:** Summary of genetic statistics of 129 *A. nanus* species at population level.

Pop	*N*	*Na*	*Ne*	*I*	*Ho*	*He*	*uHe*	F	PPB
1	25.533	6.533	4.265	1.031	0.247	0.443	0.452	0.576	73.33%
2	2.000	1.667	1.644	0.347	0.233	0.208	0.278	−0.093	33.33%
3	13.733	4.467	2.731	0.807	0.219	0.369	0.383	0.534	73.33%
4	4.000	2.333	2.145	0.543	0.217	0.292	0.333	0.355	46.67%
5	12.000	5.400	3.155	0.930	0.239	0.389	0.406	0.417	86.67%
6	17.933	9.467	4.987	1.468	0.389	0.578	0.595	0.424	86.67%
7	33.800	8.533	4.826	1.203	0.174	0.504	0.512	0.754	100.00%
8	16.867	4.400	2.857	0.922	0.134	0.458	0.473	0.727	93.33%
Mean	15.733	5.350	3.326	0.906	0.232	0.405	0.429	0.524	74.17%
SE	0.906	0.538	0.344	0.082	0.029	0.031	0.032	0.041	8.21%

**Table 8:** Pairwise matrix of Nei genetic distance of *A. nanus* populations.

Pop	1	2	3	4	5	6	7	8
1	0.000							
2	0.146	0.000						
3	0.109	0.114	0.000					
4	0.116	0.142	0.159	0.000				
5	0.084	0.141	0.169	0.124	0.000			
6	0.055	0.179	0.164	0.152	0.091	0.000		
7	0.106	0.313	0.297	0.191	0.180	0.135	0.000	
8	0.278	0.465	0.306	0.444	0.444	0.293	0.297	0.000

**Table 9:** Pairwise population Fst values of *A. nanus* populations.

Pop	1	2	3	4	5	6	7	8
1	0.000							
2	0.145	0.000						
3	0.072	0.112	0.000					
4	0.104	0.186	0.140	0.000				
5	0.061	0.096	0.096	0.076	0.000			
6	0.036	0.137	0.093	0.103	0.059	0.000		
7	0.070	0.188	0.142	0.122	0.096	0.058	0.000	
8	0.134	0.265	0.159	0.233	0.201	0.114	0.112	0.000

**Table 10:** Analysis of molecular variance (AMOVA) of *A. nanus*.

Source	DF	SS	MS	Est. Var.	Proportion
Among Pops	7	109.605	15.658	0.088	1%
Among Indiv	121	1569.546	12.971	5.662	77%
Within Indiv	129	212.500	1.647	1.647	22%
Total	257	1891.651		7.398	100%

**Table 11:** Results of bottleneck effect in *A. nanus* populations, based on SSR markers.

Pop	Wilcoxon’s Signed-Rank Test (TPM)	MODE-SHIFT
1	0.57715	L-shaped
2	0.03125	L-shaped
3	0.05371	L-shaped
4	0.03906	L-shaped
5	0.00305	L-shaped
6	0.00671	L-shaped
7	0.05536	L-shaped
8	0.63673	L-shaped

## Data Availability

The data presented in this study are available on request from the corresponding author.
